# Integrative analysis of left ventricle and epicardial adipose tissue identifies SDHA and OGDH as candidate targets for ischemic heart disease

**DOI:** 10.1016/j.isci.2026.116370

**Published:** 2026-06-12

**Authors:** Muhammad Arif, Stephen Doran, Maryam Clausen, Johannes Wikström, Mohammad Bohlooly-Y, Elias Björnson, Liam Davidsson, Anders Jeppsson, Malin Levin, Adil Mardinoglu, Jan Boren

**Affiliations:** 1 Department of Molecular and Clinical Medicine, University of Gothenburg, The Wallenberg Laboratory, Sahlgrenska University Hospital, Gothenburg, Sweden; 2 Science for Life Laboratory, University of Gothenburg, 41390 Gothenburg, Sweden; 3 Centre for Host-Microbiome Interactions, Faculty of Dentistry, Oral & Craniofacial Sciences, King’s College London, London SE1 9RT, UK; 4 Compound Synthesis & Management, Discovery Sciences, BioPharmaceuticals R&D, AstraZeneca, The Discovery Centre, Cambridge, UK; 5 Ribocure Pharmaceuticals AB, Mölndal, Sweden; 6 Centre for Genomics Research, Discovery Sciences, BioPharmaceuticals R&D, AstraZeneca, Gothenburg, 431 83 Mölndal, Sweden; 7 Science for Life Laboratory, KTH - Royal Institute of Technology, 17121 Stockholm, Sweden

**Keywords:** Cardiovascular medicine, Human metabolism, Systems biology

## Abstract

Ischemic heart disease (IHD) involves coordinated molecular changes across heart; yet, their interplay remains poorly understood. Here, we investigated transcriptomic alterations in two heart tissue subtypes, left ventricle (LV) and epicardial adipose tissue (EAT), from age- and BMI-matched healthy and IHD individuals, including diabetic and non-diabetic patients. We performed transcriptomic profiling and systems-level network analysis to identify disease-associated gene expression changes. Our analysis revealed (1) stronger transcriptional responses in EAT than LV, particularly in diabetic individuals, and (2) widespread dysregulation of inflammatory and metabolic pathways, including oxidative phosphorylation, cytokine signaling, and fatty acid degradation, across tissue subtypes. Co-expression network analysis uncovered shared gene modules, with SDHA and OGDH emerging as central, downregulated genes linked to mitochondrial function and inflammation, important processes in IHD pathophysiology. These findings were validated in independent human and mouse datasets. Overall, our integrative analysis identifies conserved molecular signatures across cardiac tissue subtypes, suggesting therapeutic potential in IHD.

## Highlights

• Matched left ventricle and epicardial adipose tissue transcriptome in ischemic heart disease
• Epicardial adipose tissue shows stronger changes, especially in diabetic patients
• SDHA and OGDH emerges as central biomarkers and targets across cardiac tissues
• Results are validated in independent public human and mouse transcriptomic data

## Graphical abstract

**Figure:**
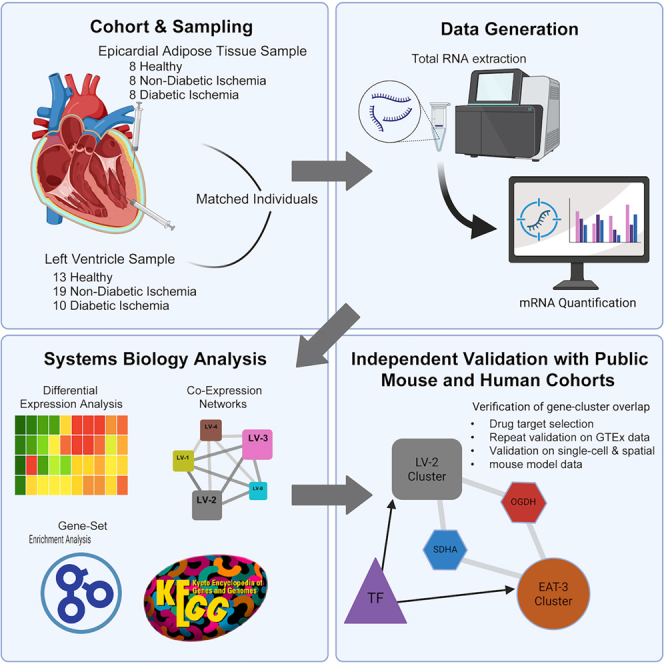

## Introduction

Ischemic heart disease (IHD) is a type of cardiovascular disorder caused by reduced blood and oxygen supply to the heart muscle. This reduction occurs due to the accumulation of plaque, particularly cholesterol deposits, within the walls of the coronary arteries, leading to their constriction. Based on data from WHO^1^ and other international and national health bodies, IHD has been identified as the primary contributor to global mortality. In 2023, the Centers for Disease Control and Prevention (CDC) projected that 5% of adults (>20 years old) have IHD in the United States, and 17% of IHD-related deaths happen in young and middle-aged adults.^2^ These numbers are likely to be underestimated as IHD is often silent and asymptomatic. A substantial portion of cases are only identified when patients experience a heart attack, a situation that frequently arises too late for effective intervention.

Despite extensive research on IHD, most transcriptomic studies have focused on a single tissue subtype such as atria or ventricles. Epicardial adipose tissue (EAT) is the visceral fat between the myocardium and the visceral pericardium and is linked to coronary events, notably in metabolic disease.^3^^,^^4^ EAT lacks a fascial barrier and shares microcirculation with the heart, enabling local paracrine effects on coronary arteries.^3^ This anatomy distinguishes EAT from pericardial fat outside the parietal pericardium.^3^ Higher EAT burden associates with incident coronary events independent of traditional risk factors^5^ and with faster coronary artery calcium (CAC) progression at follow-up.^6^ Greater EAT volume also relates to high-risk plaque features on coronary CT angiography in coronary artery disease (CAD). Note that some imaging studies quantify pericoronary adipose tissue (PCAT), the perivascular subset of EAT. PCAT attenuation reflects local perivascular inflammation rather than total EAT burden.^3^^,^^7^ In cardiometabolic cohorts, EAT burden relates to CAD severity in type 2 diabetes independent of body mass index (BMI) and CAC, and EAT volume is higher in T2D with greater atherosclerosis.^8^^,^^9^ Single-cell and single-nucleus studies of human EAT map inflammatory macrophage states along with fibro-adipogenic, endothelial, and metabolic programs relevant to coronary pathology.^10^^,^^11^ Epidemiologic links between EAT burden and coronary events are observational, so a causal effect of lowering EAT on outcomes is unproven.^3^^,^^5^ These gaps make EAT a logical focus for cross-tissue signaling studies in IHD, but coordinated profiling with other cardiac compartments, such as left ventricle (LV), remains limited.

Mitochondrial dysfunction and metabolic remodeling are recognized among the hallmarks of IHD and heart failure.^12^^,^^13^^,^^14^^,^^15^ Among the central players in these processes, succinate dehydrogenase complex flavoprotein subunit A (SDHA) and oxoglutarate dehydrogenase (OGDH) have been implicated in heart diseases. SDHA mutation has been associated to genetic heart diseases,^16^^,^^17^ while cardiac OGDH level has been linked to hypertrophic cardiomyopathy severity and proposed as a potential atherosclerosis biomarker.^18^^,^^19^ These prior findings underscore the importance of mitochondrial metabolism in IHD and support the rationale for investigating SDHA and OGDH as potential therapeutic targets.

In this study, we performed transcriptomics analysis of two distinct cardiac tissue subtypes, the LV and EAT from healthy individuals and those with IHD (Figure S1). We hypothesized that IHD induces tissue subtype- and context-specific transcriptional and network alterations, particularly in mitochondrial metabolic pathways, and that these changes are further modulated by diabetes. By comparing diabetic IHD, non-diabetic IHD, and healthy control subjects, and by analyzing LV and EAT from the same hearts, we uniquely evaluated shared and distinct molecular networks across regions and disease states to identify key drivers of IHD pathophysiology. Using systems biology and co-expression network (CN) analysis, we uncovered interactions between LV and EAT, common gene clusters, and central genes linked to disease pathology. Notably, while SDHA and OGDH have previously been implicated in cardiac mitochondrial dysfunction and metabolic remodeling, our study demonstrates their consistent emergence as central nodes across LV and EAT, in both diabetic and non-diabetic IHD. This highlights their potential as robust candidate biomarkers and therapeutic targets within regulatory networks of IHD.

## Results

### Characteristics of the study participants

This study aimed to identify transcriptional differences in the heart between healthy control and subjects with IHDs, in both diabetic and non-diabetic subjects. We recruited 14 healthy control subjects and 30 subjects with IHDs (Data S1). One control subject was excluded due to extremely high glucose level and one IHD patient was removed due to inconsistency in the sequencing data. The remaining 29 IHD patients were then split into two subgroups, the non-diabetic (ND-I, *n* = 19) and diabetic (D-I, *n* = 10) groups. We observed no significant differences in the groups’ age, body mass index, and other clinical variables, except plasma glucose, systolic blood pressure, and serum potassium level (Table 1, Data S1). As expected, the glucose level of the diabetic patients was the highest compared to other groups, together with their systolic blood pressure (Table 1).

**Table 1:** Demographic and clinical characteristics of the participants

	Healthy	Ischemia (ND-I)	Diabetic ischemia (D-I)	P-Adj (ND-I vs. healthy)	P-Adj (D-I vs. healthy)	P-Adj (D-I vs. ND-I)
N (male/female)	13 (9/4)	19 (15/4)	10 (8/2)	–		
Age (years)	67.08 ± 3.04	65.58 ± 2.01	67.90 ± 2.73	Ns	ns	ns
BMI (kg/m^2^)	27.50 ± 1.30	28.02 ± 0.95	28.04 ± 1.32	Ns	ns	ns
Glucose (mmol/L)	6.52 ± 0.32	6.80 ± 0.55	10.17 ± 1.39	Ns	<0.05	<0.05
Smoking status – *N* (percent)						
Yes	0	2 (0.11%)	1 (0.10%)	–		
No	3 (0.23%)	9 (0.47%)	1 (0.10%)			
Previously	10 (0.77%)	8 (0.42%)	8 (0.80%)			
Alcohol consumption – *N* (percent)						
Never drinks	0	3 (0.16%)	2 (0.20%)	–		
Seldom	4 (0.31%)	3 (0.16%)	5 (0.50%)			
Moderate	4 (0.31%)	7 (0.36%)	2 (0.20%)			
Frequent	3 (0.23%)	0	0			
Unknown	2 (0.15%)	6 (0.32%)	1 (0.10%)			
Snus – *N* (percent)						
Yes	1 (0.08%)	0	3 (0.30%)	–		
No	11 (0.85%)	12 (0.63%)	6 (0.60%)			
Previously	1 (0.08%)	2 (0.11%)	1 (0.10%)			
Unknown	0	5 (0.26%)	0			
History of cardiovascular problems – *N* (percent)						
Angina pectoris	0	18 (0.95%)	9 (0.90%)	–		
Aortic valve insufficiency	1 (0.08%)	0	0			
Aortic stenosis	11 (0.85%)	0	1 (0.10%)			
Congenital	1 (0.08%)	0	0			
Arrhythmia	1 (0.08%)	2 (0.11%)	0			
Hereditary CVD	3 (0.23%)	7 (0.37%)	4 (0.40%)			
History of medications – N (percent)						
Currently medicated	12 (0.92%)	19 (1.00%)	10 (1.00%)	–		
Diuretic	3 (0.23%)	5 (0.26%)	3 (0.30%)			
ACE-inhibitor	2 (0.15%)	10 (0.53%)	4 (0.40%)			
Beta-blocker	8 (0.62%)	14 (0.74%)	10 (1.00%)			
CA-antagonist	2 (0.15%)	6 (0.32%)	4 (0.40%)			
Anti-arrhythmia	0	1 (0.05%)	0			
Lipid inhibitor	8 (0.62%)	15 (0.79%)	10 (1.00%)			
ASA	6 (0.46%)	15 (0.79%)	9 (0.90%)			
Warfarin	1 (0.08%)	2 (0.11%)	0			
Clopidogrel	0	1 (0.05%)	0			
Nitro	1 (0.08%)	10 (0.53%)	3 (0.30%)			
Bronchodilator	2 (0.15%)	1 (0.05%)	0			
Other drugs	6 (0.46%)	6 (0.32%)	8 (0.80%)			

From the remaining 42 subjects (13 healthy control and 29 IHD), we collected left ventricular (LV) tissue. In addition, from a subset of 24 subjects (*n* = 8 per group), we also collected matched EAT. In total, this resulted in 66 transcriptomic samples (42 LV and 24 matched EAT). Transcriptomic profiling was performed on these samples (Data S1).

### Left ventricle of ischemic subjects shows a significant increase in inflammatory responses and a decrease in carbohydrate metabolism pathways

**Figure 1:**
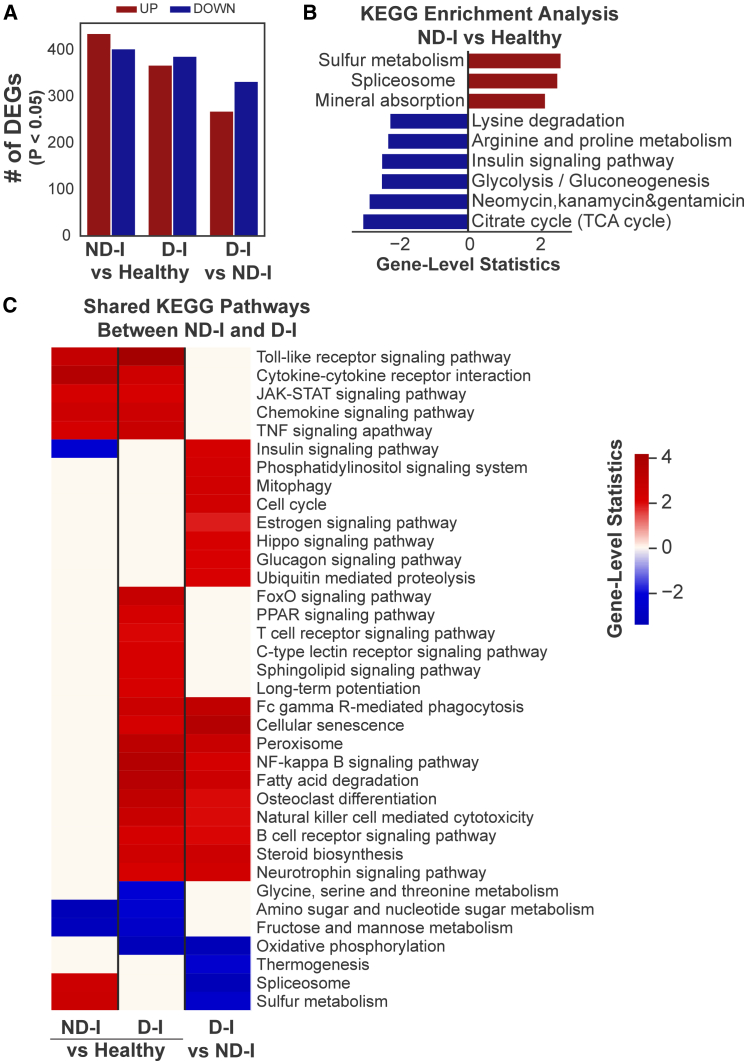
Transcriptomics of heart LV (A) The differential expression analysis showed alterations in significant transcriptional profile alterations (*p* value <0.05) between the ischemic (ND-I and D-I) and healthy subjects. (B) KEGG pathways (*p* value <0.05) that were uniquely altered when comparing ND-I with healthy subjects. (C) Shared KEGG pathways (FDR <0.01) were significantly altered when comparing D-I subjects with healthy and ND-I subjects.

First, we performed a differential expression analysis of the LV transcriptomics data (Data S2). We identified 839 (436 up- and 403 down-regulated) and 755 (368 up- and 387 down-regulated) differentially expressed genes (DEGs) when comparing ND-I and D-I groups to healthy subjects, respectively (Figure 1A). To understand the affected biological functions associated with these transcriptomics changes, we performed gene-set enrichment analysis (GSEA) to identify the association of the DEGs with biological pathways and processes (Data S2). The GSEA using KEGG pathways in ND-I subjects showed up-regulation of sulfur metabolism, spliceosome, and mineral absorption pathways compared to the healthy group (Figure 1B). On the other hand, we observed significant down-regulation in several metabolic pathways in ND-I samples compared to healthy subjects, specifically lysine degradation, arginine and proline metabolism, insulin signaling, glycolysis/glucogenesis, and tricarboxylic acid cycle (TCA) cycle. We observed bigger changes in the pathways when comparing D-I samples with healthy control (Figure 1C). Specifically, inflammatory and cell death pathways were up-regulated, e.g., NF-κB signaling, natural killer cell signaling, Fc gamma R-mediated phagocytosis, B and T cell receptor signaling pathways, and cellular senescence, while oxidative phosphorylation pathway was down-regulated. We also observed the up-regulation of pathways associated with lipid metabolism (PPAR signaling, sphingolipid biosynthesis, and steroid pathways) and fatty acid degradation, and the down-regulation of glycine, serine, and threonine metabolisms. Also, we observed similarities in the alteration caused by both conditions. Both conditions showed down-regulation of carbohydrate metabolism pathways and up-regulation of important inflammation and immune response pathways, specifically TNF, chemokine, JAK-STAT, cytokine-cytokine receptor, and Toll-like receptor signaling pathways (Figure 1C). We further confirmed these alterations by performing GSEA on gene ontology biological process and observed similar changes in the associated terms (Figure S2).

We also compared the D-I subjects with ND-I. There were 269 up- and 333 down-regulated genes in this comparison (Figure 1A). Although many of the altered pathways were similar to D-I vs. healthy comparisons, we observed that several pathways were uniquely altered in the D-I compared to ND-I subjects (Figure 1C), including the up-regulation of phosphatidylinositol, estrogen, hippo, and glucagon signaling pathways, and cellular processes (mitophagy and cell cycle), while thermogenesis was down-regulated.

### Ischemic epicardial adipose tissue exhibits more prominent transcriptional alterations compared to the left ventricle

**Figure 2:**
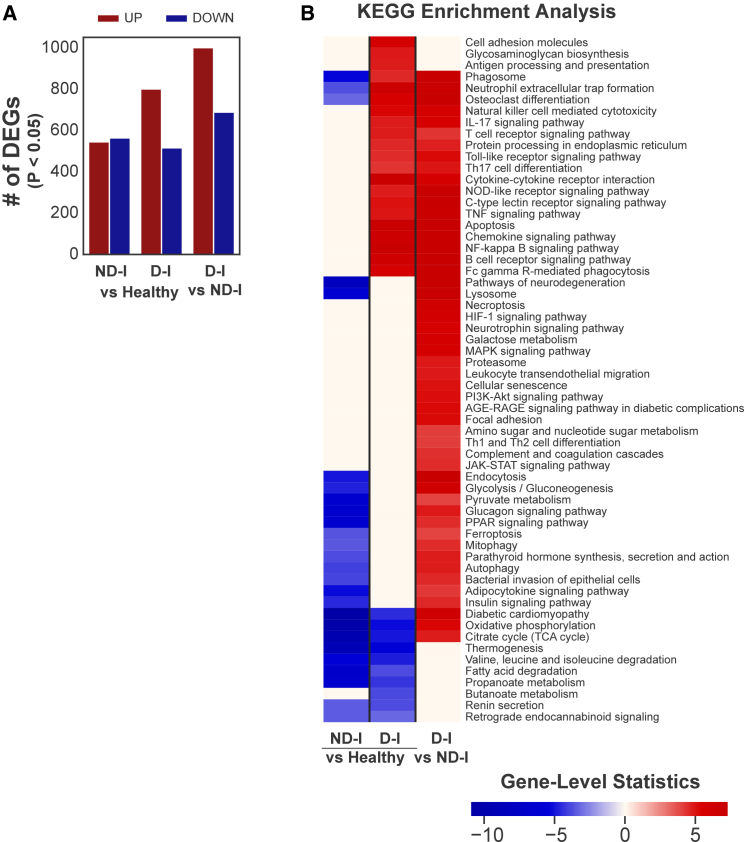
Transcriptomics of EAT (A) The differential expression analysis showed alterations in significant transcriptional profile alterations (*p* value <0.05) between the ischemic (ND-I and D-I) and healthy subjects. (B) KEGG pathways (FDR <0.01) that are uniquely altered when comparing ND-I and D-I with healthy subjects, and ND-I vs. D-I.

We performed a similar analysis for the EAT transcriptomics data (Data S3) and, interestingly, observed significantly higher DEGs compared to LV, especially in D-I subjects. We identified 1,109 (545 up- and 564 down-regulated) and 1,317 (801 up- and 516 down-regulated) DEGs in ND-I and D-I subjects when compared to the healthy group (Figure 2A). Functionally, EAT showed a more pronounced effect in their response to IHD (Figure 2B and Data S3). In ND-I subjects, we observed more down-regulated metabolic pathways associated with lipids, amino acids, carbohydrates, and other important signaling pathways. The altered metabolic pathways in LV were also found to be down-regulated in EAT (Figure 2B). The D-I subjects showed similar, but more pronounced, up-regulation of inflammatory and cell death pathways as LV (Figure 2B). Moreover, we observed a fibrosis-related pathway, cell adhesion molecule, to be up-regulated in D-I subjects compared to healthy, while the butanoate metabolism pathway was down-regulated (Figure 2B).

We also identified multiple commonly regulated pathways in both ND-I and D-I groups when compared to healthy subjects (Figures 2B and S3). Mitochondrial functions and oxidative phosphorylation were down-regulated, together with diabetic cardiomyopathy and thermogenesis pathways. Moreover, we identified several metabolic pathways to be commonly down-regulated in both conditions, such as the TCA cycle, valine, leucine, and isoleucine degradation, fatty acid degradation, propanoate metabolism, and retrograde endocannabinoid signaling pathways. Interestingly, we observed that many known fibroproliferative pathways and processes, such as extracellular matrix organization and osteoclast differentiation, were up-regulated in the D-I group but down-regulated in the ND-I group, together with antigen processing and presentation and neutrophil-related immune response (Figures 2B and S3).

Furthermore, we compared D-I to ND-I subjects and observed high dysregulation in transcriptomics data of EAT (1,001 up- and 689 down-regulated genes) (Figure 2A). Functional analyses showed similar dysregulation as D-I and healthy comparison (Figure 2B), several pathways were uniquely significantly up-regulated in D-I compared to ND-I subjects, including signaling pathways (HIF-1, MAPK, PI3K-Akt, and JAK-STAT), immune (Th1 and Th2 cell differentiation and leukocyte transendothelial migration), focal adhesion, and cellular senescence. This showed that, although both have similar disease etiology, D-I subjects have higher biological alterations compared to ND-I subjects.

### Gene co-expression network unveils tissue subtype-specific clusters associated with ischemic heart diseases

In the previous sections, we successfully identified the transcriptional alterations and affected biological pathways in response to IHDs in both LV and EAT. To further study the molecular functional relationships and identify driver pathways associated with the disease, we employed CNs. CNs are a powerful tool in untangling the complexity of big biological data in healthy and diseases,^20^ including cardiovascular,^21^ fibrotic,^22^ and metabolic diseases.^23^ Here, we generated two tissue subtype-specific CNs (LV and EAT) and performed downstream network analysis on the top 25% significantly positively co-expressed genes (FDR <0.05) in each network. We employed the Leiden community detection algorithm^24^ to better understand the network by identifying clusters with distinct network structures. Furthermore, we calculated the average transitivity of the clusters to identify the most central clusters in each network.^25^ We hypothesized that the most central clusters play important roles in responding to the disease, thus their gene members are rational therapeutic target candidates.

**Figure 3:**
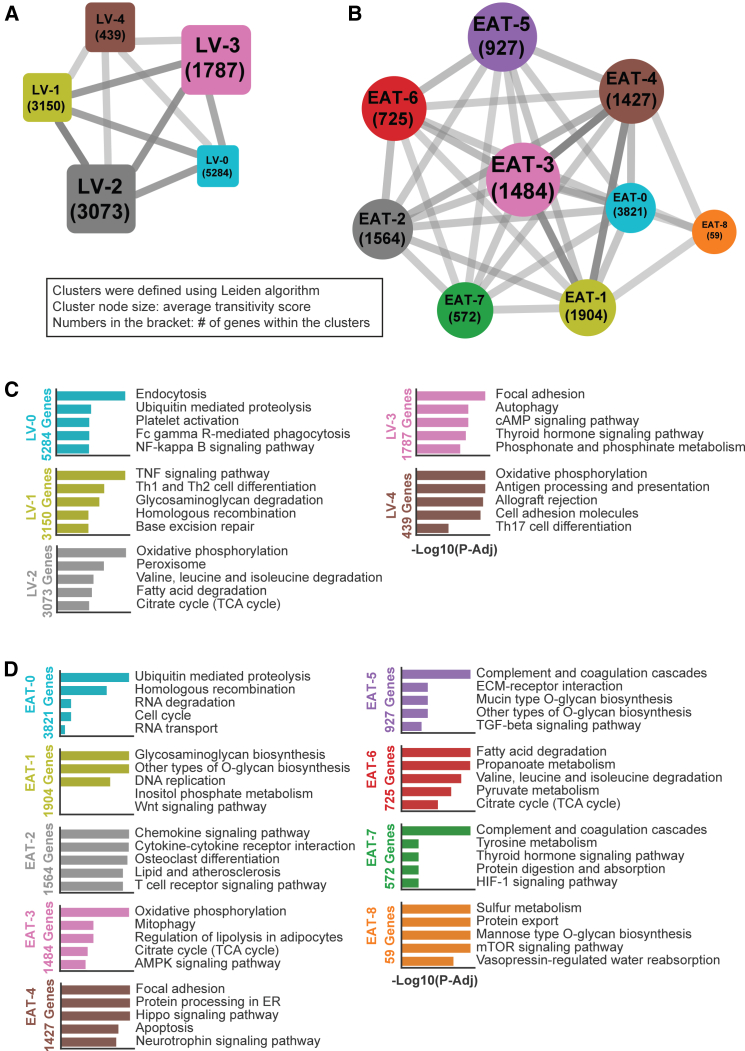
Heart tissue subtype-specific CNs (A) LV-specific CN consisted of five clusters with distinct gene members with LV-2 and LV-3 as the two most central clusters, based on their average transitivity scores (node size). The numbers below the name denote the number of genes within the clusters. (B) EAT-specific CN consisted of nine clusters with EAT-3 and EAT-5 as the two most central clusters. (C) Top five associated KEGG pathways of each cluster of LV-specific CN. (D) Top five associated KEGG pathways of each cluster of EAT-specific CN.

Using the LV transcriptomics data, we generated an LV-specific CN with 13,733 gene nodes connected with 1.6 M undirected edges. From the CN, we identified five distinct clusters (Figure 3A) with the biggest cluster, LV-0, containing 5,284 genes and the smallest, LV-4, with 439 genes. Interestingly, based on their average transitivity (Data S4), we found LV-2 (3,073 genes) and LV-3 (1,787) as the central and driver clusters of the LV-specific CN. Similarly, we generated EAT-specific CN. It has 12,934 gene nodes connected with more than 258 K undirected edges and identified nine distinct clusters (Figure 3B) with EAT-0 and EAT-8 as the biggest and smallest clusters, with 3,821 and 59 genes, respectively. EAT-3 and EAT-5 showed the highest average transitivity; thus, we defined them as the most central and driver network in EAT-specific CN.

To further understand the cluster roles in the disease, we performed GSEA with KEGG Pathways on each cluster to identify the functions associated with their gene members (Figures 3C and 3D). Interestingly, the central clusters from both tissue subtype-specific CNs also showed top similar functional roles. LV-2 and EAT-3 were enriched with genes associated with oxidative phosphorylation. Moreover, the genes from both clusters were also associated with an important metabolic pathway, the TCA cycle, and pathways associated with lipid metabolism, specifically peroxisome and fatty acid degradation in LV-2 and regulation of lipolysis in adipocytes in EAT-3. Uniquely, LV-2 was enriched with genes associated with valine, leucine, and isoleucine degradation (Figure 3C) and EAT-3 with mitophagy and AMPK signaling pathways (Figure 3D). The other central clusters, LV-3 and EAT-5 were enriched with fibrosis-related pathways: focal adhesion and extracellular matrix (ECM)-receptor interaction (Figures 3C and 3D). LV-3 was also enriched with genes related to autophagy, cAMP signaling, thyroid hormone signaling, and phosphonate and phosphinate metabolism pathways (Figure 3C), and EAT-3 with complement and coagulation cascades, glycan biosynthesis, and TGF-beta signaling pathway (Figure 3D).

### Reporter metabolite analysis of CN connects central clusters to NAD metabolism and ubiquinone and ubiquinol metabolites

**Figure 4:**
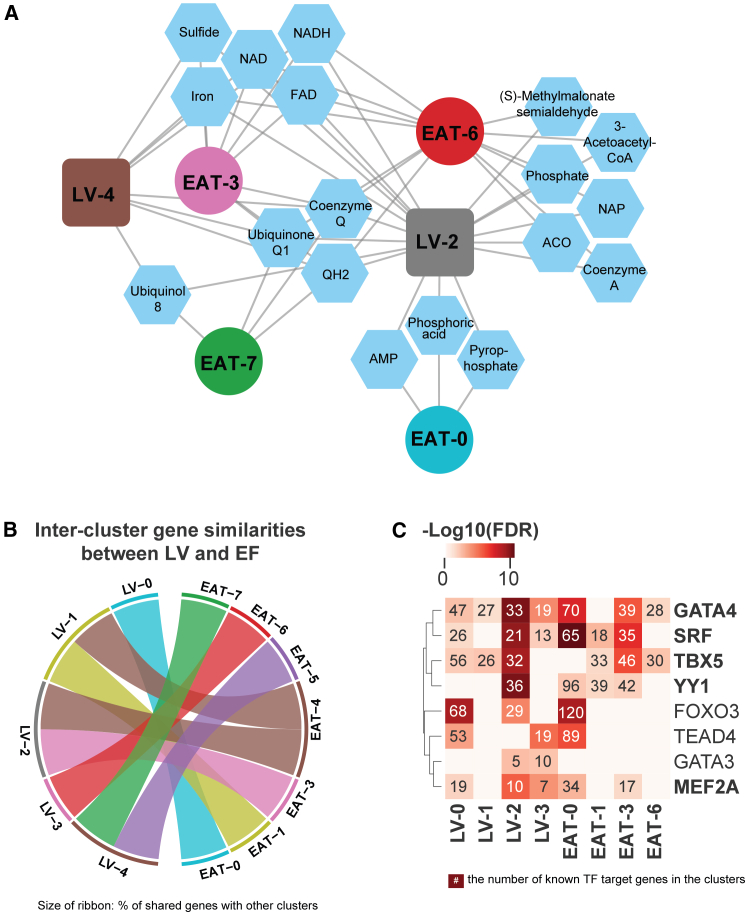
Reporter metabolites and commonalities between LV and EAT-specific CN clusters (A) Reporter metabolites analysis results connected to their associated CN clusters. Only metabolites and clusters with degree centrality >1 were shown. For a more complete figure, refer to Figure S4. (B) Hypergeometric test results revealed the inter-cluster gene similarities between LV and EAT-specific CNs. (C) Known cardiac TFs and their regulated clusters in both regions CNs. The annotations denoted the number of known TF target genes from the clusters. Bold gene names denoted the TFs regulating both LV-2 and EAT-3.

Since the functional analyses from differential expression and network analyses showed a high association between IHD and metabolic pathways, we performed reporter metabolite analysis on each cluster to understand the cluster gene members association with metabolites. We found 828 metabolites significantly associated with LV-0, LV-2, LV-4, EAT-0, EAT-3, EAT-6, and EAT-7 (Figure S4). We further isolated the metabolites that were associated with at least two clusters to examine the clusters’ commonalities (Figure 4). We focused on LV-2 and EAT-3, as these were the only central clusters with significantly associated reporter metabolites. By far, LV-2 showed the highest connectivity with 819 metabolites and shared them with all other clusters (Figure S4). On the other hand, EAT-3 is only connected to eight metabolites (Figure S4), and all of them are also connected to LV-2 (Figure 4), including important metabolites such as NAD, NADH, and FAD. Moreover, they were also connected to sulfide and iron, known central metabolites of sulfur metabolism and mineral absorption, respectively. Finally, we observed that gene members of LV-2 and EAT-3 were also associated with coenzyme Q, ubiquinone Q1, and QH2 (ubiquinol) (Figure 4A).

### LV-2 and EAT-3 are regulated by known cardiac transcription factors and exhibit potential as the location of therapeutic targets

Based on the functional and reporter metabolite similarities of the central clusters described above, we assumed that the CN of these two distinct heart tissue subtypes was driven by similar genes. To validate that, we performed the hypergeometric test to identify these similarities (Data S4). We found that indeed LV-2 and EAT-3 shared a significant number of genes (Figure 4B) but not between LV-3 and EAT-5. Based on the facts that LV-2 and EAT-3 were both identified as central clusters and shared important genes and pathways related to the diseases, we hypothesized that one or more shared genes in LV-2 and EAT-3 might be the rational therapeutic targets to treat IHDs, for both diabetic and non-diabetic patients.

To identify candidate therapeutic targets, first, we were interested in the common transcription factors (TFs) that regulate LV-2 and EAT-3. We performed reporter TFs analysis^26^ on all clusters (Data S4) and successfully mapped 170 TFs to the 14 LV and EAT clusters (Data S4). We identified 68 TFs that are commonly associated with LV-2 and EAT-3 clusters (Figure S5). Interestingly, out of the eight known cardiac TFs that we successfully mapped to the clusters, five of them were mapped, among others, to both LV-2 and EAT-3, namely GATA4, SRF, TBX5, YY1, and MEAT2A (Figure 4C).

### Identification of candidate therapeutic targets for ischemic heart diseases

Based on the previous analyses, although we had two distinct tissue subtypes of heart, we successfully showed the similarities in their response to IHD. Moreover, these analyses supported the hypothesis that gene members of the central clusters, specifically LV-2 and EAT-3, may represent potential targets for mitigating the effects of IHD. To pinpoint the target candidates, we narrowed down the common genes in LV-2 and EAT-3 and examined if they are associated with the shared reporter metabolites or known cardiac TFs, mentioned in the previous sections, and we found 20 genes (Data S5) that fulfilled all those criteria. To facilitate pre-clinical research, we examined the expression changes of these genes in three mouse myocardial infarction (MI) publicly available data^21^^,^^27^^,^^28^ and found four genes to be differentially expressed in all cohorts: SDHA, OGDH, SDHC, and FLAD1. We decided to focus on SDHA and OGDH as they are also FDA-approved drug targets, based on the information from the Human Protein Atlas (https://www.proteinatlas.org/).^29^ SDHA and OGDH were both the target of two main known cardiac TFs, SRF and TBX5. Furthermore, OGDH was associated with two reporter metabolites, NAD and NADH, while SDHA is known to be associated with the rest of the reporter metabolites.

**Figure 5:**
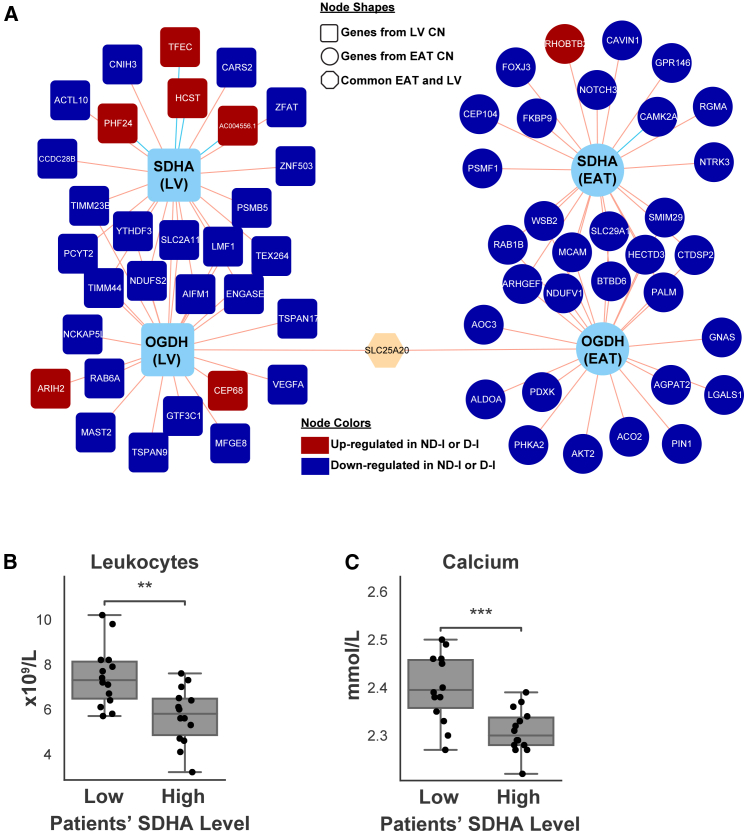
SDHA as a candidate therapeutic target for IHD (A) Network representation of genes directly connected to SDHA within LV- and EAT-specific CN. Node shapes indicate tissue specificity (LV-specific, EAT-specific, or shared between LV and EAT, while colors reflect the direction of DEGs of ND-I or D-I versus healthy (red, up-regulated; blue, down-regulated). Central nodes highlight SDHA and OGDH in each tissue, illustrating tissue-specific and shared regulatory contexts. (B) Peripheral leukocyte counts (×10^9^/L) stratified by low versus high SDHA expression in LV tissue, demonstrating significantly altered inflammatory status associated with reduced SDHA levels. (C) Serum calcium concentrations (mmol/L) in subjects with low versus high LV SDHA expression, indicating a significant association between SDHA expression and systemic calcium homeostasis. (Boxplots show median, IQR, 1.5× IQR whiskers, and outliers. *t* test, ∗∗ *p* < 0.01, ∗∗∗ *p* < 0.001).

To further understand their functional roles, we isolated SDHA, OGDH, and their top ten neighbors from LV and EAT-specific networks and their shared neighbors (Figure 5A) that were significantly altered in ND-I or D-I compared to healthy patients. In LV, we observed that SDHA and OGDH were a direct neighbor (high correlation) and shared 11 neighboring genes that were down-regulated in ND-I or D-I, including AIFM1 (associated with apoptosis), PCYT2 and LMF1 (lipid metabolism), TIMM23B and TIMM44 (mitochondria), PSMB5 (proteasome), ENGASE and SLC2A11 (carbohydrate metabolism), and NDUFS2 (mitochondria, NADH, and FAD activity, also diabetic cardiomyopathy). Interestingly, when we checked their shared neighbors in EAT, we found a unique set of 11 down-regulated genes, including MCAM (vascular wound healing), RAB1B (glycoprotein metabolism), NDUFV1 (mitochondria), and ARHGEF7 (known to be related to cardiac arrest and coronary heart disease). The functional relevance of SDHA’s and OGDH’s neighbors to cardiovascular diseases (CVDs) further reinstates their potential role in IHD pathology.

To further understand the systemic effect of SDHA, we re-stratified LV and EAT transcriptomics samples to low and high SDHA (bottom and top 1/3 or 33.3 percentile, respectively) and compared the important clinical variables (Data S1) between low and high SDHA groups. We found that the subjects with low LV SDHA levels have significantly higher leukocyte and calcium (Figures 5B and 5C). High levels of these clinical variables are known to be associated with higher inflammations, metabolic dysfunctions, and cardiovascular problems. These correlations further emphasized the importance of the proposed genes to cardiovascular and physiological health.

### Low cardiac SDHA and OGDH level are associated with adverse outcomes of ischemic heart disease

**Figure 6:**
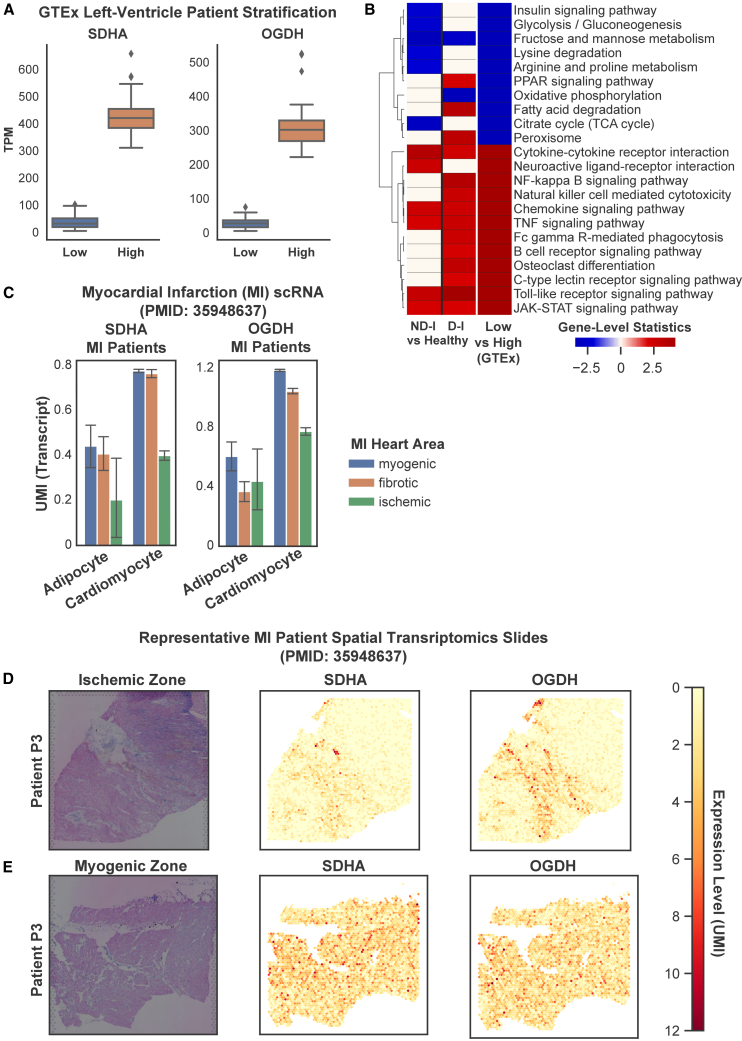
SDHA and OGDH in publicly available heart LV data (A) TPM of low and high SDHA and OGDH levels from heart LV GTEx data. (Boxplots show median, IQR, 1.5× IQR whiskers, and outliers). (B) Functional analysis of subjects with high SDHA and OGDH compared to the low group. (C) The UMI expression of SDHA and OGDH in the single-cell transcriptomics data of MI patients in the two highest expressing cell types: adipocytes and cardiomyocytes. (Bars represent the mean, and error bars represent 95% confidence intervals). (D) Representative spatial transcriptomics expression of SDHA and OGDH from an MI patient (P3) from ischemic and (E) myogenic zone.

To test our hypothesis on the central role of SDHA and OGDH in the LV and EAT-specific CNs and their association with important clinical variables, we used publicly available heart LV data from GTEx. We selected subjects older than 60 years old, to replicate the population from our study (Table 1) and stratified them to low and high SDHA and OGDH (bottom and top 1/3 or 33.3 percentile of transcripts per million (TPM) values, respectively, for both genes) with 41 samples in high and 47 in the low group (GTEx ID of the selected samples can be found in Data S6). The high group consisted of 27 male and 14 female subjects with an average SDHA TPM value of 432.35 ± 11.13 (mean ± SEM) and OGDH of 311.07 ± 10.69 (Figure 6A). Meanwhile, the low group consisted of 33 male and 14 female subjects with an average SDHA TPM value of 38.39 ± 3.69 and OGDH of 27.29 ± 2.29 (Figure 6A). We performed differential expression and functional analysis between the low and high groups and found that many pathways that were altered in ND-I and D-I subjects compared to healthy were worsened in the low SDHA and OGDH group (Figure 6B). We observed the down-regulation of metabolic pathways, including glycolysis, fructose and mannose metabolism, lysine degradation, fatty acid degradation, and TCA cycle, and other important pathways, including oxidative phosphorylation and peroxisome (Figure 6B). Moreover, similar trends were followed by many mitochondrial-related biological processes (Data S6). Meanwhile, pathways and biological processes related to inflammatory response were up-regulated, including cytokine-cytokine receptor interaction, TNF, NF-κB, chemokine, JAK-stat, and B cell receptor signaling pathways, natural killer cell-mediated toxicity, and Fc gamma R-mediated phagocytosis (Figure 6B and Data S6).

### Single-cell and spatial transcriptomics data show SDHA and OGDH cell-specificity and emphasizes their involvement in the pathophysiology of ischemic heart disease

By highlighting the central role of SDHA and OGDH in the LV- and EAT-specific CNs, and their association with important clinical variables and pathways in IHD, our analysis demonstrated the strength of network-based approaches to capture meaningful biological signal in heterogeneous patient population, which might be missed by differential expression analysis. This has also been demonstrated in previous studies.^30^^,^^31^ In line with this, we observed modest down-regulations in both LV and EAT of ND-I and D-I compared to healthy (*p* > 0.05), with a similar trend in our validation cohorts. We hypothesized that this attenuation is due to sample heterogeneity (e.g., disease severity, lifestyle, dietary intake, and medications), as we observed significant down-regulations of SDHA and OGDH in bulk transcriptomics data in three controlled mouse studies.^21^^,^^27^^,^^32^ To further test our hypothesis, we retrieved publicly available human heart single-cell data that included healthy and MI subjects.^33^

We observed that SDHA was expressed (unique molecular identifier or UMI >0) mainly in cardiomyocytes (70.1% of healthy and 60.4% of MI) and then followed by adipocytes (26.7% in healthy and 33.1% in MI) (Figure S6). Focusing on those cell types, we observed similar small down-regulation in SDHA levels as in bulk transcriptomics data, around 16.4% decrease in cardiomyocyte in MI compared to healthy (0.736 ± 0.003, mean ± SEM of UMI per cell vs. 0.881 ± 0.005), and 10% decrease in adipocytes (0.397 ± 0.029 vs. 0.441 ± 0.199) (Figure S6). But when we investigated deeper in ischemic compared to the non-ischemic (myogenic) heart zone of the MI patients, we observed much bigger down-regulation in the SDHA level in almost all cell types (Figure 6C). Specifically, we observed a 48% decrease in cardiomyocytes (0.400 ± 0.011 vs. 0.776 ± 0.003 for ischemic and myogenic, respectively) and 54% in adipocytes (0.442 ± 0.091 vs. 0.203 ± 0.047). Interestingly, the SDHA level in other necrotic areas of the heart, specifically the fibrotic area, was only decreasing by 1.6% in cardiomyocytes (0.764 ± 0.009) and 7.9% in adipocytes (0.407 ± 0.038) (Figure 6C). Similar trends were observed with the expression of OGDH, where we observed a 34% decrease in cardiomyocytes (0.775 ± 0.013 vs. 1.187 ± 0.004 for ischemic and myogenic, respectively) and 27% in adipocytes (0.400 ± 0.108 vs. 0.607 ± 0.050) (Figure 6C). Moreover, in the fibrotic area, the expression of OGDH decreased by 11% in cardiomyocytes (1.046 ± 0.009) and 39% in adipocytes (0.369 ± 0.034) (Figure 6C). These observations were validated by spatial transcriptomics data from one of the patients (P3) from the same cohort, where we observed lower SDHA and OGDH expression in the ischemic area (Figure 6D) compared to the myogenic area (Figure 6E).

## Discussion

IHD is a complex and systemic disease, affecting millions of people in the world. In this study, we generated transcriptomics and employed an extensive systems and network biology analysis to generate a comprehensive transcriptomics profile of IHD. One unique feature of our study was that we collected not only heart LV, but also matching EAT from age and BMI-concordant healthy and ischemic subjects. Another unique feature is the inclusion of well-characterized diabetic and non-diabetic ischemic patients based on their clinical records and serum chemistry (i.e., glucose level) data. Subsequently, we generated transcriptomics data and performed differential expression and functional analysis to identify heart-tissue subtype-specific transcriptional and biological changes associated with the disease, including inflammatory and metabolic pathways. Furthermore, we leveraged CN analysis to identify driver clusters of each tissue subtype and explore their shared regulatory patterns. Through these analyses, we identified SDHA and OGDH as key genes and potential therapeutic targets and biomarkers for IHDs. To emphasize the relevance of SDHA and OGDH to the disease, we stratified heart transcriptomics data from GTEx to high and low SDHA and OGDH groups and compared them. Finally, we validated our proposed targets in previously published mouse and human single-cell and spatial transcriptomics data.

When comparing healthy and ischemic subjects, we observed EAT exhibited more pronounced transcriptional changes compared to LV in response to the disease. This increase is particularly notable in diabetic patients. This observation implies that EAT could serve as a more effective tissue subtype for assessing cardiovascular health and monitoring ischemia progression, particularly in diabetic patients, consistent with established literature.^3^^,^^34^^,^^35^ Nonetheless, we found many similar affected pathways and functions between the two tissue subtypes, especially for diabetic subjects. Our functional analyses, consistent with the literature,^36^^,^^37^^,^^38^ revealed significant up-regulation of inflammatory pathways, such as cytokine-cytokine receptor interaction, Toll-like receptor, chemokine, NF-κB, and TNF signaling, in both regions. Similarly, we also observed down-regulation in oxidative phosphorylation and metabolic pathways, specifically amino acid and carbohydrate metabolism pathways, in both regions. Interestingly, we detected an opposite regulation of the fatty acid degradation pathway in the LV and EAT, which suggests an interplay between the regions as noted in the literature.^39^^,^^40^

Our CN analyses provided additional emphasized these shared characteristics and interplay between the two regions. We showed that the driving forces behind the LV and EAT networks consisted of genes associated with crucial biological pathways including oxidative phosphorylation, TCA cycle, lipid metabolism, amino acid metabolism, and fibrosis-related pathways, i.e., focal adhesion and ECM-receptor interaction. These pathways have been studied extensively due to their significance in the pathogenesis of IHD.^41^^,^^42^^,^^43^^,^^44^ Additionally, according to reporter metabolite analysis, the central network clusters were both enriched with genes linked to NAD and ubiquinol-related metabolites. Notably, reporter TF analysis indicated that these central clusters also featured target genes of established cardiac TFs.

Another significant result of our CN analysis was the discovery of two promising candidate biomarkers and therapeutic targets: SDHA and OGDH. Both genes were situated in the central clusters (LV-2 and EAT-3), linked to critical ischemia-related functions and pathways, and regulated by known cardiac TFs (SRF and TBX5). Moreover, they are heart-enhanced based on the Human Protein Atlas. Furthermore, we validated their expression down-regulations in multiple different independent cohorts in mice and humans^21^^,^^27^^,^^28^^,^^33^ using bulk, single-cell, and spatial transcriptomics data. Additionally, it is worth noting that both genes are FDA-approved and safe druggable targets.

SDHA is a key component of mitochondrial complex II that is essential in the electron transport chain, energy generation, and TCA cycle. Specifically, it is involved in the conversion of succinate to fumarate, FAD to FADH2, and the transfer of electrons from succinate to ubiquinone.^45^ SDHA has been implicated in multiple diseases, including different cancer types,^46^ mitochondrial disorders,^47^ and Leigh syndrome.^48^ SDHA mutation has also been linked to genetic heart diseases^16^^,^^17^ and dilated cardiomyopathy.^49^ Moreover, using our serum and plasma chemistry data, we observed that subjects with low SDHA have significantly higher leukocyte and calcium compared to the high group. High leukocyte has been used as a signal for internal inflammation in the body and its increase has been linked to the severity of CVDs.^50^^,^^51^^,^^52^ Similarly, the use of calcium supplementation, which implies elevating the level of calcium in the body, is also associated with a higher risk of cardiovascular mortality.^53^^,^^54^

OGDH functions as a rate-limiting enzyme in the TCA cycle, specifically in the conversion of 2-oxoglutarate to succinyl coenzyme A, and tryptophan metabolism. Recent publications showed a significant decrease in cardiac OGDH levels in subjects with hypertrophic cardiomyopathy.^18^ An obesity-related pre-clinical study showed that the OGDH ± mice have higher body and liver weight, and heightened susceptibility to liver dysfunction when subjected to high-fat diets.^55^ Other publications showed the increase of OGDH and citrate synthase levels in the adrenal gland were proposed as potential biomarkers for atherosclerosis under chronic stress^19^ and the involvement of OGDH in several cancer, developmental, and neurological disorders.^56^^,^^57^^,^^58^^,^^59^

However, the roles of SDHA and OGDH in IHD are still relatively unknown. To illuminate their potential roles, we stratified the heart transcriptomics data from GTEx data to high and low SDHA and OGDH groups. Our analysis unveiled notable patterns: within the low SDHA and OGDH groups, pathways associated with inflammation exhibited up-regulation in the low group compared to the high. These pathways include cytokine-cytokine receptor interaction, NF-κB, chemokine signaling, TNF signaling, Toll-like receptor signaling, and JAK-STAT signaling pathways. Conversely, metabolic pathways were down-regulated in the low group, including lysine and fatty acid degradation, TCA cycle, fructose and mannose metabolism, and glycolysis/glucogenesis. In summary, this analysis underscored the exacerbation of vital pathways impacted by IHD due to low SDHA and OGDH expression. This suggests a potential role for SDHA and OGDH as potential therapeutics aimed at alleviating the effects of IHD.

In summary, our study provides valuable insights into the molecular landscape of IHD and unveils promising candidates for further investigation and development of therapeutic interventions. We employed systems biology and data-driven approaches to systematically study the molecular, specifically transcriptional, alterations and dysregulation caused by the disease, in both diabetic and non-diabetic subjects. We also used CN analysis to identify central clusters and genes associated with the disease. Finally, we identified SDHA and OGDH as potential biomarkers and therapeutic targets to attenuate the damage caused by the disease, highlighting their crucial roles in the pathogenesis of IHD.

### Limitations of the study

Although our study offers valuable insights, it is important to acknowledge certain limitations. First, our analysis heavily depended on transcriptomics data, potentially constraining the depth of our findings’ sensitivity. Additionally, our analyses focused on protein-coding genes, overlooking the substantial impact of IHD on non-protein coding genes, including microRNAs, long non-coding RNAs, and circular RNAs, which are known to regulate key pathological processes such as inflammation, vascular remodeling, fibrosis, and cardiomyocyte survival.^60^^,^^61^ Several non-coding RNAs have been shown to act as disease biomarkers and modulators of cardiovascular pathology, suggesting that their exclusion may limit the ability to capture important regulatory layers of disease-associated transcriptomic changes. This study also relied on the correlational relationship between the genes and the disease, not causal relationships. Finally, we acknowledge that, in the present study, several clinical variables that may influence transcriptomic profiles were unavailable, including the localization of obstructed vessels, the number of affected vessels, and the duration of diabetes. Future investigations hold considerable potential to enhance this study’s scope by encompassing other omics (e.g., metabolomics, proteomics, and metagenomics) in both bulk and single cell level, with more detailed patient information, and non-protein coding genes in their exploration.

## Resource availability

### Lead contact

Further information and requests for resources should be directed to and will be fulfilled by the lead contact, Muhammad Arif (muhammad.arif@gu.se).

### Materials availability

This study did not generate new unique reagents; the reagents used are available commercially.

### Data and code availability

• The processed transcriptomics data (raw counts and TPM), together with non-identifiable clinical and demographic data can be retrieved from Data S1.
• The code for the analysis and visualization (grouped based on the figure numbers) is available at https://github.com/muharif/2025.Arif_Doran_etal_IschemicHeartDisease.
• The individual-level RNA-seq data generated in this study are sensitive human genomic/transcriptomic data and cannot be openly deposited because of GDPR restrictions and the risk of participant re-identification. Data sharing is governed by applicable legal, ethical, and institutional requirements, including GDPR, Swedish ethical review legislation, the informed consent, and University of Gothenburg procedures for sensitive personal data.
• Controlled-access deposition of the restricted individual-level data is planned through FEGA Sweden, the Swedish node of the Federated European Genome-phenome Archive. Submission is currently pending finalization of institutional procedures. No EGA accession number is yet available.
• A public metadata record describing the dataset is available at the SciLifeLab Data Repository (https://doi.org/10.17044/scilifelab.c.8474868) and will be updated with EGA accession information once deposition is complete. Until then, qualified researchers may direct data access enquiries to the lead contact. Requests will be evaluated by the University of Gothenburg in accordance with applicable legal and ethical requirements. No direct or private transfer of restricted individual-level data can be provided outside approved controlled-access procedures.

## Acknowledgments

This work was supported by the SciLifeLab & Wallenberg Data Driven Life Science Program (M.A., grant, KAW 2020.0239). The computations and data handling were enabled by resources provided by the National Academic Infrastructure for Supercomputing in Sweden (NAISS), partially funded by the 10.13039/501100004359Swedish Research Council through grant agreement no. 2022-06725.

## Author contributions

M.A.: methodology, software, validation, formal analysis, investigation, data curation, writing – original draft, visualization, and funding acquisition; S.D.: methodology, formal analysis, data curation, and writing – original draft; M.C.: data curation, resources, and writing – review and editing; J.W.: data curation, resources, and writing – review and editing; M.B.-Y.: data curation, resources, and writing – review and editing; E.B.: investigation, data curation, and writing – review and editing; L.D.: visualization and writing – review and editing; A.J.: resources and writing – review and editing; M.L.: investigation, supervision, and writing – review and editing; A.M.: supervision and writing – review and editing; J.B.: investigation, supervision, writing – review and editing, and funding acquisition. M.A. and S.D. contributed equally to this project.

## Declaration of interests

M.C. and M.B.-Y. are employees of AstraZeneca, Gothenburg, Sweden and J.W is an employee of Ribocure Pharmaceuticals AB, Mölndal, Sweden. The rest of the authors declare no conflict of interest.

## Declaration of generative AI and AI-assisted technologies in the writing process

During the preparation of this manuscript, the authors used ChatGPT (OpenAI) for spelling and grammar assistance. The authors carefully reviewed and revised the content and accept full responsibility for the accuracy and integrity of the final publication.

## STAR★Methods

### Key resources table

**Table:** 

REAGENT or RESOURCE	SOURCE	IDENTIFIER
**Deposited data**		

RNA-Seq Data	this paper	Federated EGA (Record Metadata https://doi.org/10.17044/scilifelab.c.8474868)
Clinical and Demography Data	this paper	Data S1
RNA-Seq Data	Arif et al.^21^	GSE153485
RNA-Seq Data	Williams et al.^28^	GSE104187
RNA-Seq Data	Ounzain et al.^27^	GSE52313
Single-Cell and Spatial Transcriptomics	Kuppe et al.^33^	https://zenodo.org/record/6578047

**Software and algorithms**		

Python 3.7	Python Package	RRID:SCR_019053
R	R Project for Statistical Computing	RRID:SCR_001905
Kallisto	Bray et al.^62^	RRID:SCR_016582
Rpy2	Python Package	https://rpy2.github.io/
DESeq2	R Package	RRID:SCR_015687
PIANO	R Package	RRID:SCR_003200
SciPy	Python Package	RRID:SCR_008058
Statsmodel	Python Package	RRID:SCR_016074
iGraph	Python Package	RRID:SCR_019225
Leiden Clustering	Python Package	https://github.com/vtraag/leidenalg
Statsmodel	Python Package	RRID:SCR_016074
Gseapy	Python Package	https://gseapy.readthedocs.io/en/latest/
Enrichr	Python Package	RRID:SCR_001575
Analysis code	Python	https://github.com/muharif/2025.Arif_Doran_etal_IschemicHeartDisease

### Experimental model and study participant details

#### Sample collections from human subjects

Cardiac ischemic biopsies were obtained from 30 patients undergoing coronary artery bypass surgery at the Sahlgrenska University Hospital, Gothenburg, Sweden, due to significant atherosclerotic blockage in the epicardial coronary arteries. The surgery and sample collections were performed between 7 February 2013 and 1 February 2017. All patients were examined through echocardiography prior to operation, and the ejection fraction (EF) was measured. Non-ischemic biopsies from the left ventricle were obtained from 14 subjects undergoing aortic valve replacement in the same hospital, with angiography verified absence of coronary artery disease in any of the major myocardial coronary artery branches. Individual level data on age, sex, and other information is available in Data S1. All biopsies were collected from the left ventricle septum region with a 1 mm needle. The average weight of collected tissues samples was 100 mg and these were stored at -80°C until analysis. All patients gave informed and written consent. Patient characteristics (including diabetes status) were either collected as the patients entered the study or retrieved later from their medical charts. The diagnoses were based on ICD-codes from patient records and national databases. The study was approved by the Gothenburg Regional Ethics Committee and done according to the Declaration of Helsinki (Dnr 064-14).

### Method details

#### RNA extraction and sequencing

Total RNA was isolated from biopsies relating to 44 samples from the left ventricle using RNeasy Fibrous Tissue Mini kit (QIAGEN) and 24 samples from epicardial fat using RNeasy Lipid Tissue Mini kit (QIAGEN). These 68 samples were processed with SMARTer® Stranded RNA-Seq Kit (Takara Bio) for reverse transcription, generation of double stranded cDNA and subsequent library preparation. All libraries were quantified with the Fragment Analyzer using the standard sensitivity NGS kit (Agilent Technologies), pooled in equimolar concentrations and quantified with a Qubit Fluorometer (ThermoFisher Scientific), the library pool was further diluted and sequenced using 150 cycles on an Illumina NextSeq500.

### Quantification and statistical analysis

#### Clinical and demography data analysis

The clinical and demography data were compared using one-way ANOVA (*“f_oneway”*) with Tukey’s post-hoc (*“pairwise_tukeyhsd”*) test. All of these analysis modules were provided by the *Statsmodel*^63^ v0.13.2. We used the *“ttest_ind”* function when comparing these variables in the low and high SDHA and OGDH groups (Figures 5B and 5C) from SciPy^64^ package version 1.7.3. Variables with q-values <0.05 were considered significant in both analyses (∗∗: *p* < 0.01; ∗∗∗: *p* < 0.001). Boxplots show median, IQR, 1.5× IQR whiskers, and outliers.

#### Transcriptomics data analysis

The paired-end RNA-sequencing raw files were quantified using Kallisto^62^ with an index file generated from the Ensembl human reference genome^65^ and mapped to gene-level count and TPM using the mapping from Ensembl Biomart website, by selecting only the protein-coding transcripts and genes. Differential expression and functional analysis were performed using an in-house *DESeq2*^66^ and Piano^67^ wrapper in Python, using the *rpy2* version 3.2.2 package. The multiple hypothesis testing was done within the packages with Benjamini-Hochberg correction. We used *seaborn*^68^ version 0.11.2 package to visualize the results. The KEGG and Gene Ontology biological processes gene set collections were retrieved from *Enrichr*,^69^^,^^70^ specifically the “Human 2021” version.

#### Co-expression network analysis

Co-expression networks were generated using Spearman correlation rank, from *SciPy* version 1.7.3 package,^64^ in each tissue subtype using the TPM count data, after removing the bottom 25% of the least varying genes. Gene-gene correlation with (1) Benjamin-Hochberg FDR <0.05 and (2) among the top 25% positively correlated genes was kept for further analysis (“*multipletests*” function from Statsmodel v0.13.2). Network analyses were done with iGraph^71^ version 0.9.11 and community detection/clustering analysis was done using Leiden^24^ algorithm in iGraph. *Enrichr*^69^^,^^70^ was used for the clusters’ functional analysis.

#### Single-cell and spatial transcriptomics visualization

The single-cell and spatial transcriptomics data were retrieved from their respective data repository and the pre-processed h5ad files were loaded using *scanpy*^72^ version 1.9.3 package, without any further processing. The UMI bar plots were generated using the *Seaborn* version 0.11.2 package. Barplots show mean and the error bars represent 95% confidence interval.

## Supplemental information

Document S1. Figures S1–S6

Data S1. Unprocessed data underlying the analysis and items in the manuscript, including demography, clinical, and transcriptomics data, related to figures 1–7

Data S2. Differential expression and functional analysis results for LV transcriptomics data, related to Figures 2 and S1

Data S3. Differential expression and functional analysis results for EAT transcriptomics data, related to Figures 3 and S2

Data S4. Clusters information, reporter metabolites, inter-subtype cluster hypergeometric test, and reporter transcription factor analysis results for each cluster in LV and EAT-specific network, related to figures 3, 4, S3, and S4

Data S5. Selection criteria and information on the shortlisted candidate genes, related to Figure 5

Data S6. GTEx sample list, their SDHA and OGDH level stratification and differential expression and functional analysis from Low vs. High comparison, related to Figure 6
