# Supplementary material for: Integrative analysis of left ventricle and epicardial adipose tissue identifies SDHA and OGDH as candidate targets for ischemic heart disease
Source: iScience. 2026 Jun 12;29(7):116370. doi: 10.1016/j.isci.2026.116370 (PMC13276791; doi:10.1016/j.isci.2026.116370)
Supplement: Document S1. Figures S1–S6 [file mmc1.pdf]

iScience, Volume *29*

Supplemental information

Integrative analysis of left ventricle and epicardial adipose tissue identifies SDHA and OGDH as candidate targets for ischemic heart disease

Muhammad Arif, Stephen Doran, Maryam Clausen, Johannes Wikström, Mohammad Bohlooly-Y, Elias Björnson, Liam Davidsson, Anders Jeppsson, Malin Levin, Adil Mardinoglu, and Jan Boren

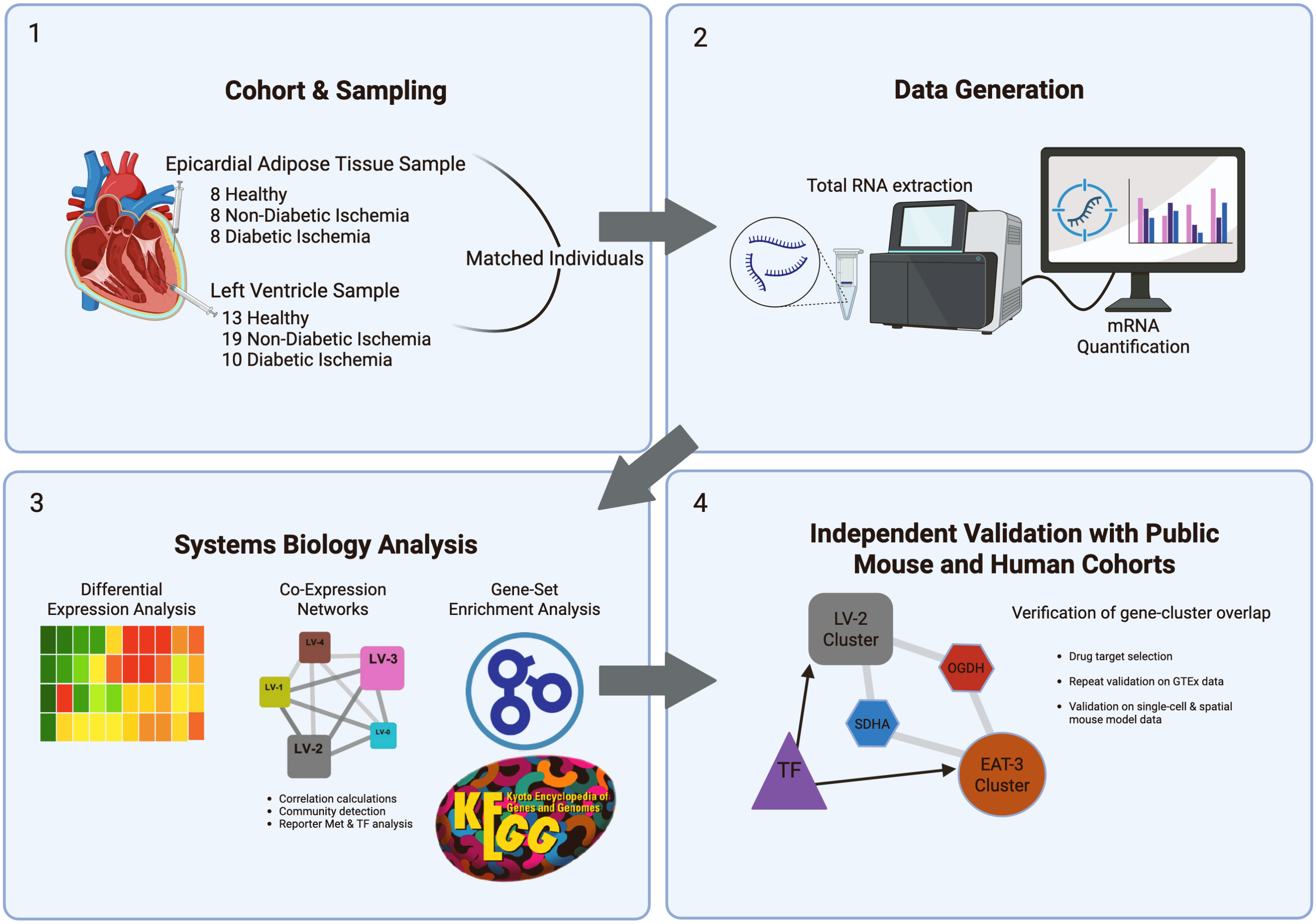

Figure S1: Graphical Abstract and Study Flow

Commonly Regulated GO Biological Process (Left Ventricle)

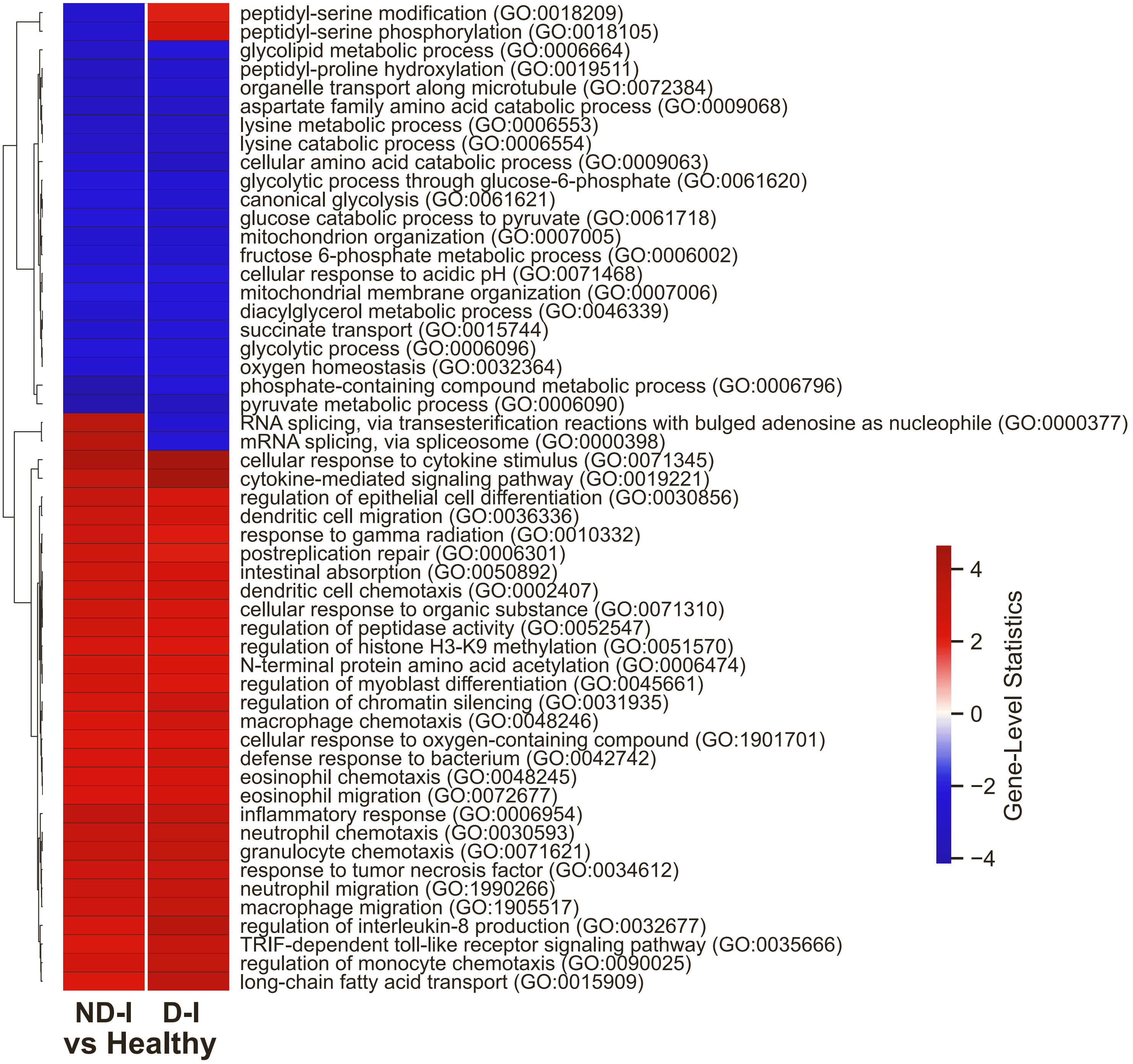

Figure S2: Commonly altered gene ontology biological process (P-Value < 0.01) between ischemic (ND-I and D-I) and healthy groups from Heart Left Ventricle transcriptomics data (Related to Figure 1).

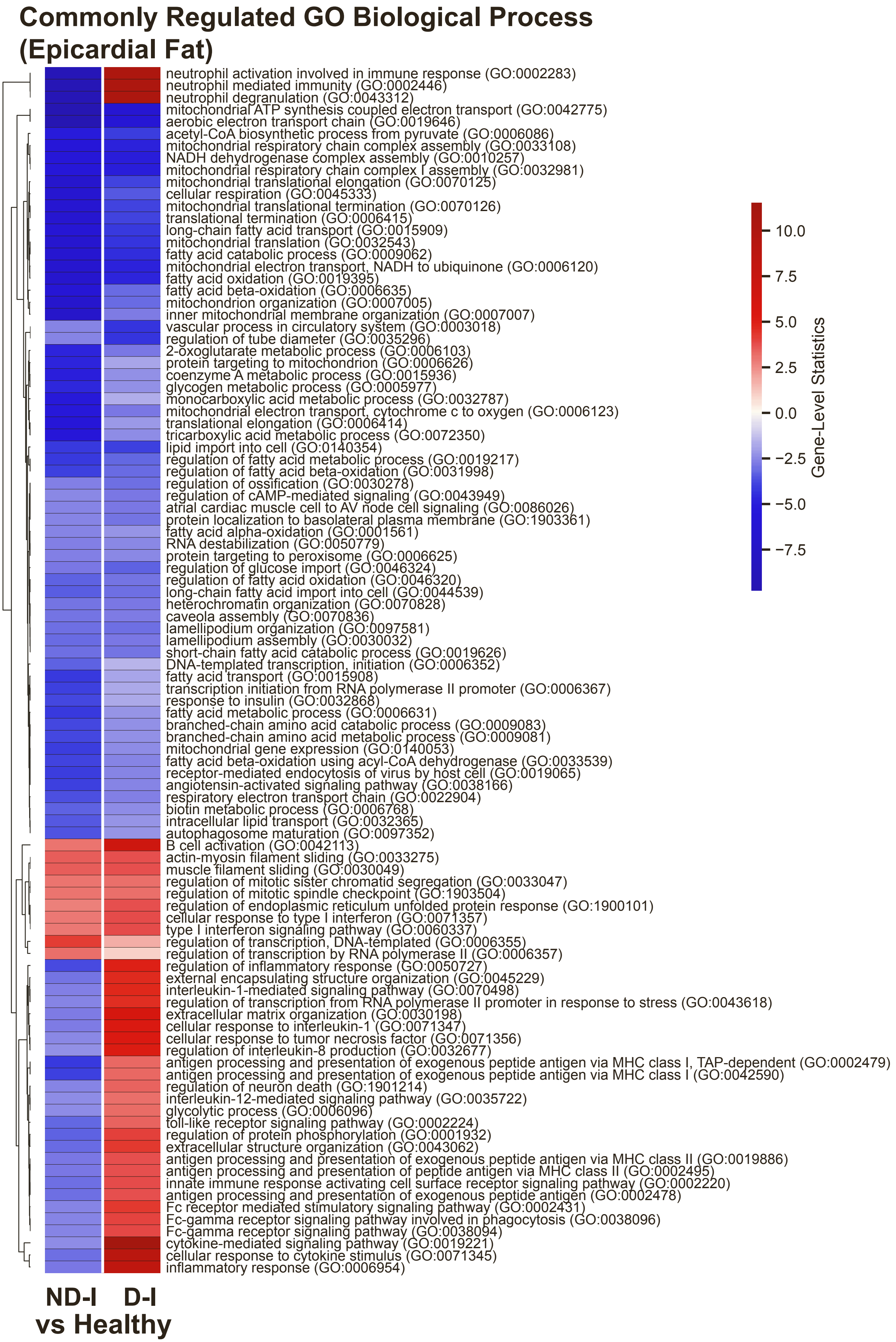

Figure S3: Commonly altered gene ontology biological process (P-Value < 0.01) between ischemic (ND-I and D-I) and healthy groups from Epicardial Adipose TIssue transcriptomics data (Related to Figure 2).

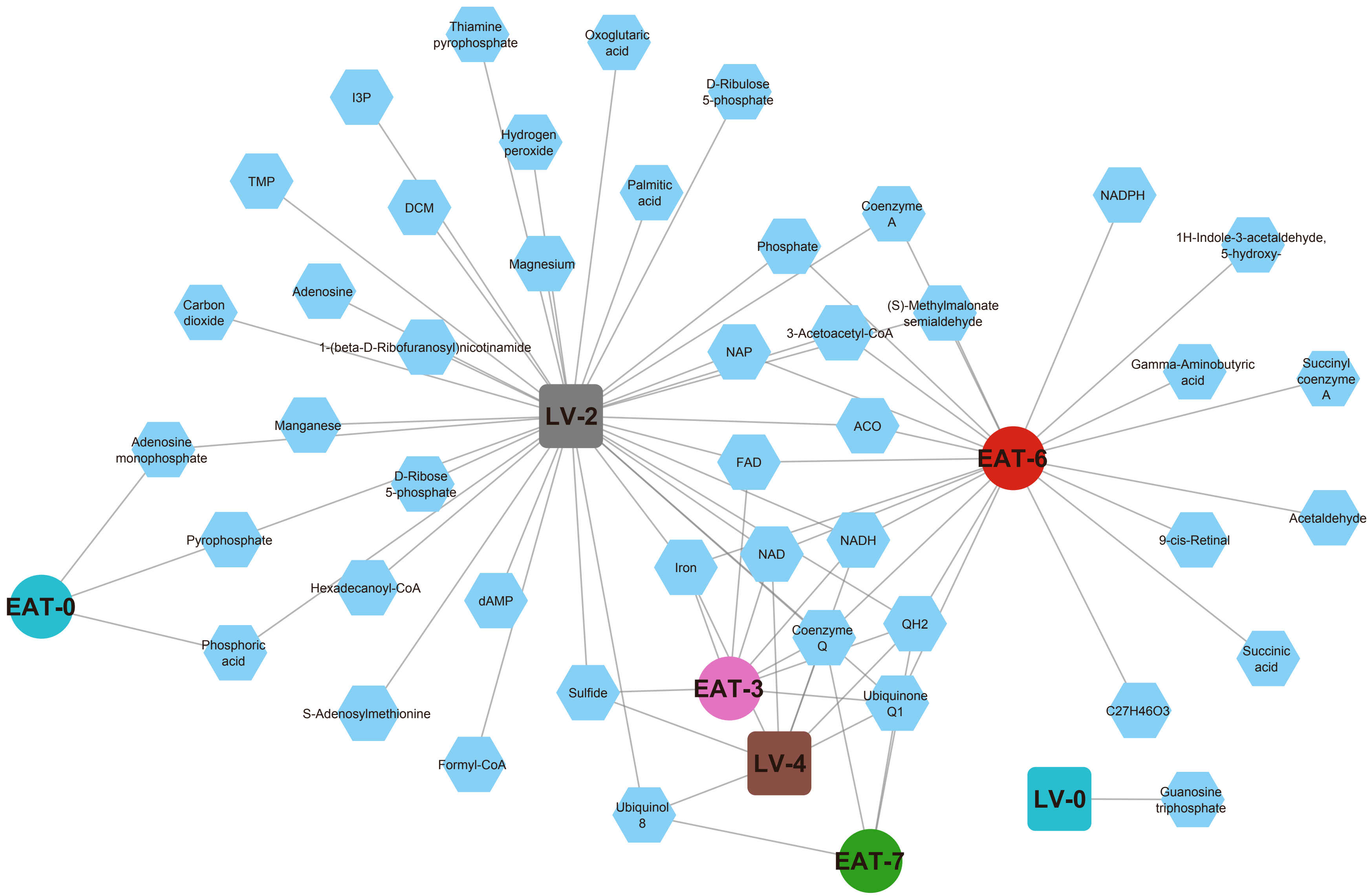

Figure S4: Reporter metabolite analysis results connected to their associated CN clusters. For visualization purposes, we removed 763 diacylglycerol subtypes and 21 unnamed metabolites that were connected to LV-2 (Related to Figure 4)

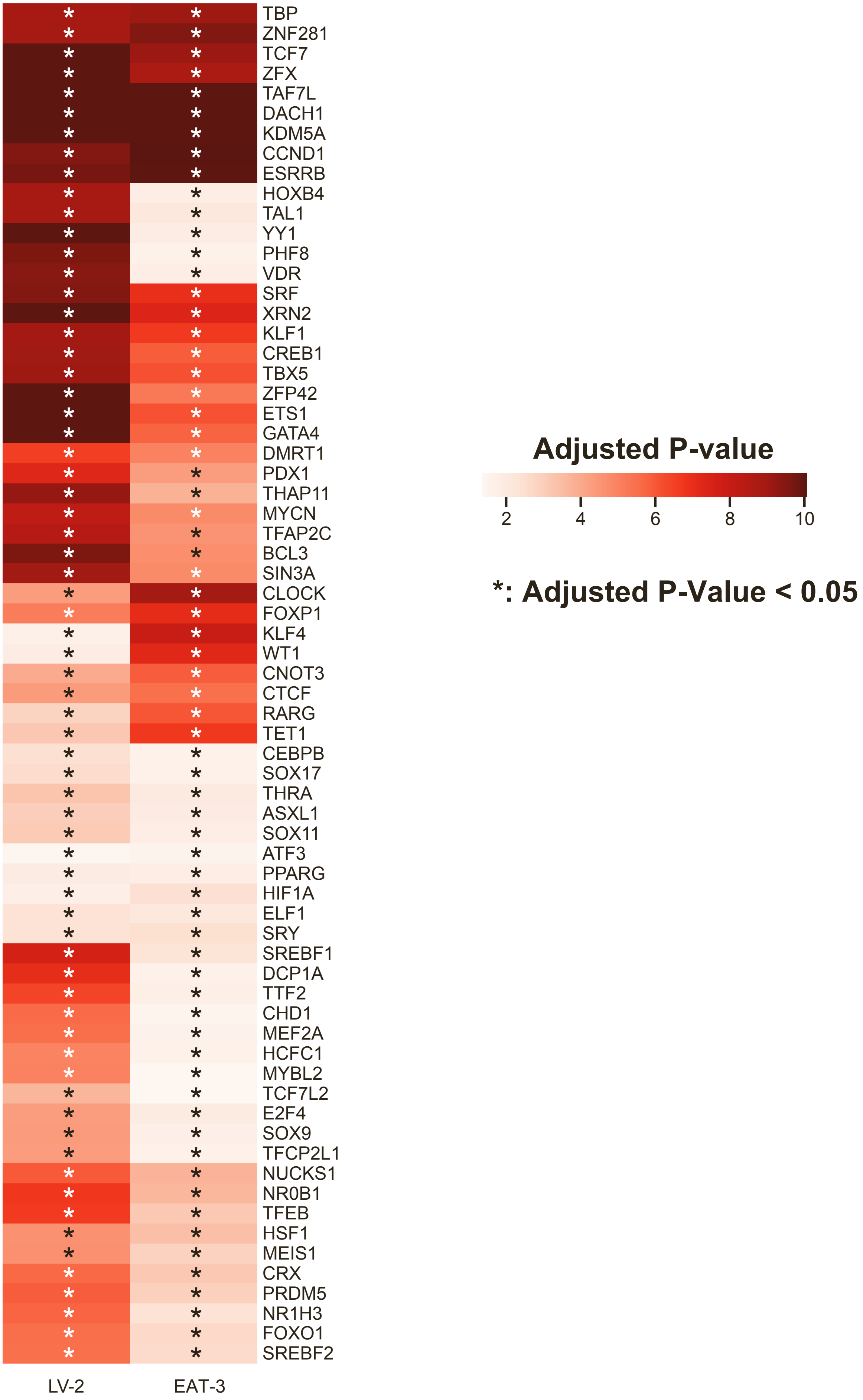

Figure S5: Common reporter transcription factor (TF) results between LV-2 and EAT-3. The annotations denoted the number of known TF target genes from the clusters (Reporter analysis with PIANO, *: Adjusted P-Value < 0.05, Related to Figure 4).

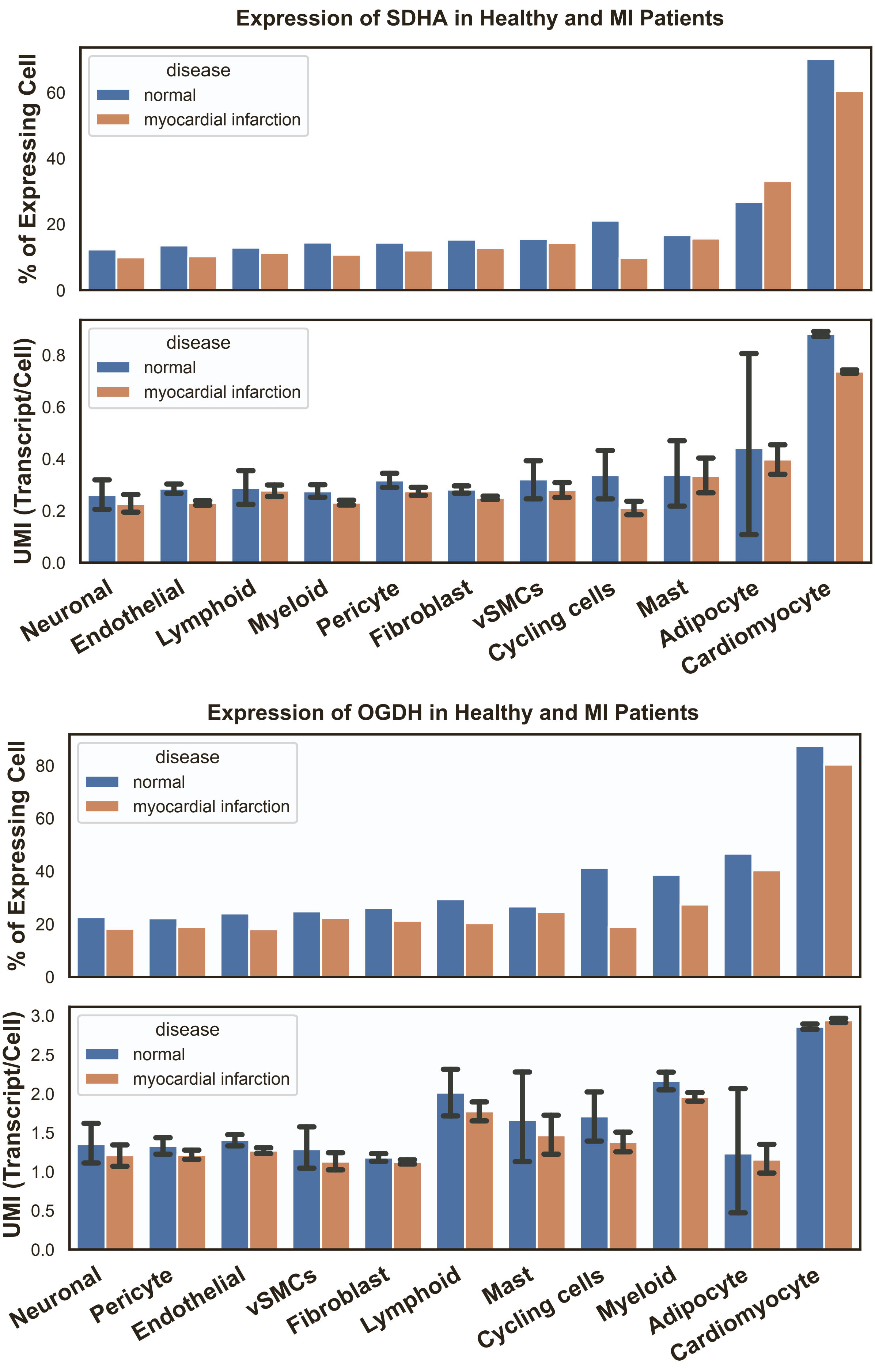

Figure S6: Single-cell expression of SDHA and OGDH when comparing in healthy and myocardial infarction heart (Bars represent the mean, and error bars represent 95% confidence intervals, Related to Figure 6).
